# The Laryngeal Affections of Voice-Users

**Published:** 1908-08-15

**Authors:** 


### Laryngology and Rhinology.

### THE LARYNGEAL AFFECTIONS OF VOICE-USERS.

The conditions under which the voice is used in different occupations are very various, and produce diverse laryngeal lesions, which form an important group among the cases of "occupation-disease." The ill-effects may involve any of the tissues of the larynx: epithelium, connective-tissue, glands, vessels, or muscles. Thus one may find simple thickening of the cords or definite pachydermia; laryngeal nodules or fibromata; hypersecretion or dryness; acute hyperæmia, chronic congestion or submucous hæmorrhage; and paresis of the muscles, varying from weakness of the internal tensors to functional inactivity of any or all the adductor muscles.

In singing, especially, the smoothest working and most perfect control of the larynx are essential; it therefore follows that a departure from the normal condition of health, too slight to be detected with the laryngoscope, may nevertheless seriously interfere with the function of the organ. Whilst any of the lesions mentioned above may follow any form of voice-use, there is a tendency for certain vocal occupations to produce special ill-effects. Thus singers are generally more or less well trained in voice-production, and they use the voice for short periods at a time and at definite degrees of pitch: they are subject to subacute hyperæmia of the cords, lasting for a shorter or longer period after singing, to increased secretion or undue dryness of the larynx, and to weakness of the internal tensors of the cords; but they are not so liable to grosser lesions such as pachydermia or laryngeal nodules. Another important class employ the voice for longer periods at a time, but with long intervals between. These are preachers and public speakers; they are saved by their intervals of rest from hypertrophic changes, but their voices are too often untrained and they frequently have to speak under conditions of emotional and intellectual excitement. They are especially apt to suffer from paresis of the muscles and from such neurotic disturbances as laryngeal spasm on attempting to speak. On the other hand, school-teachers may be considered representative of the class who use the voice for many hours at a time daily and without a sufficient period of rest; they are liable to chronic congestion and to such hypertrophic changes as laryngeal nodules and fibromata. It may be noted that the former, though generally called "singer's nodules," are not very common in trained singers, but are much more often seen in school-teachers: still they are also common among the unskilled vocalists of village choirs. Those who employ the voice violently, with no attempt at skilful production, such as auctioneers and street-hawkers, though liable to any of these lesions, may also sustain more violent accidents, such as submucous hæmorrhage; the latter also show pachydermia, but probably alcohol and syphilis are more important factors in its causation than is the use of the voice.

These affections are caused far more by misuse than by over-use of the voice. With the exception of singers, very few professional voice-users attempt to learn how to employ the most delicate of all musical instruments. There is in this one advantage, that treatment is the more likely to be successful, for a period of rest followed by a course of training in voice-production may be expected to produce a good result. On the other hand, it is not easy for the medical attendant to offer advice on the method of voice-production to a trained vocalist. Next to faulty methods of production, use of the voice during a catarrh is a frequent cause of failure. Any unhealthy condition of the air-passages above the larynx is most important; nasal obstruction not only, by producing mouth-breathing, predisposes to catarrh, but also diminishes the proper resonance of the voice, makes correct production impossible, and demands a greatly increased effort from the larynx. The general health is also a very important factor; the larynx is far more easily injured if the general health be poor. Menstruation has a marked effect on the larynx, and is often accompanied by a tendency to congestion; a vocal effort at this period is certainly more likely to damage the larynx.

In the treatment, therefore, of these conditions, every attention must be given to improving the conditions under which the voice is used, to the method of production, to the state of the nasal passages and naso-pharynx, and to the general health. Vocal rest is the great curative agent, but when the organ is restored to normal the work must be resumed under improved conditions, or the trouble will recur. Direct local and general medication is of far less importance. If the laryngeal muscles are acting weakly, strychnine is indicated, and in cases of chronic catarrh small doses of potassium iodide or of the green iodide of mercury are usually recommended. Locally, a stimulating application in a steam-inhaler, such as vapor pini sylvestris, or a similar drug applied with a nebuliser, is of some value. Painting the larynx with astringents is not advisable unless there are marked hypertrophic changes. Under such treatment singer's nodules will frequently disappear without operation, and, even when the forceps have been used, a similar course is afterwards necessary to prevent recurrence.

